# Associations of perfluoroalkyl substances (PFAS) with terminal ductal lobular unit involution of the normal breast

**DOI:** 10.1186/s13058-025-02103-9

**Published:** 2025-08-18

**Authors:** Katherine W. Reeves, Youssef Oulhote, Philippe Grandjean, Flemming Nielsen

**Affiliations:** 1 Department of Biostatistics & Epidemiology, University of Massachusetts Amherst, Amherst, MA USA; 2 Department of Environmental Medicine and Public Health, Mt. Sinai Icahn School of Medicine, Icahn School of Medicine at Mount Sinai, New York, USA; 3 National Institute of Public Health, University of Southern Denmark, Odense, Denmark; 4 Department of Public Health, University of Southern Denmark, Odense, Denmark

**Keywords:** Per- and polyfluoroalkyl substances (PFAS), Involution, Terminal ductal lobular unit

## Abstract

**Background:**

Per- and polyfluoroalkyl substances (PFAS) may be carcinogenic, and animal studies demonstrate their harmful effects on mammary gland development. Terminal ductal lobular units (TDLUs) are the structures that produce milk following childbirth, and involution of TDLUs normally occurs with aging. Most breast cancers arise from TDLUs, and a greater degree of TDLU involution may be associated with lower breast cancer risk. We estimated associations between PFAS concentrations and TDLU involution in normal breast tissue samples.

**Methods:**

Concentrations of seven PFAS were measured in serum provided by a subset of 263 cancer-free volunteer participants from the Susan G. Komen for the Cure Tissue Bank (KTB) who were postmenopausal, not currently using hormone therapy, and had available TDLU measurements made by a trained pathologist examining H&E stained section of a core biopsy sample of tissue from the outer upper quadrant of a single breast. Bayesian kernel machine regression and quantile-G computation were used to estimate covariate-adjusted associations between the PFAS mixture and measures of TDLU involution (presence of TDLUs, number of observed TDLUs, and median TDLU span) within this population and with stratification on parity and breastfeeding history.

**Results:**

TDLUs were observed in breast tissue samples of 40.3% (*N* = 106) of the study population, with similar PFAS concentrations between participants with and without observed TDLUs. No strong, statistically significant associations were observed between individual PFAS and presence of observed TDLUs. The overall effect of the PFAS mixture suggested an inverted U-shaped association with odds of observed TDLUs, although this was not statistically significant (β = 0.03 95% CI -2.75, 2.81; *p* = 0.98). Among the subgroup of parous women, stratified analyses suggested a positive association between the PFAS mixture and observed TDLUs among those who had ever breastfed, but a slightly negative association among those who had never breastfed.

**Conclusions:**

Overall, our analysis does not support meaningful effects of PFAS on TDLU involution, although we note that these findings are not applicable to premenopausal women or to postmenopausal women using hormone therapy.

**Supplementary Information:**

The online version contains supplementary material available at 10.1186/s13058-025-02103-9.

## Background

Per- and polyfluoroalkyl substances (PFAS) have detrimental effects on the mammary gland in experimental studies [1–5]. International Agency for Research on Cancer identifies the two most common PFAS, perfluorooctanoic acid (PFOA) and perfluorooctane sulfate (PFOS), as carcinogenic and possibly carcinogenic, respectively [6]. PFAS are widely used in non-stick, water-resistant, and stain-resistant coatings for consumer products. Biomonitoring studies report detectable levels of PFAS in > 98% of the U.S. population [7]. PFAS accumulate in human tissues and may have half-lives of many years [8–10], prompting major concerns about their potential for long-term health effects. Although not genotoxic [11], they may interact with peroxisome proliferator-activated receptor-α and also interact with steroid hormones [11]; these mechanisms are particularly concerning as each may stimulate carcinogenic processes.

Accumulating evidence suggests that PFAS exposure could promote breast cancer. Experiments using mouse models show that perfluorooctanoic acid (PFOA) exposure delays mammary gland development [2]. Separate studies showed that these effects can persist across three generations [3, 5] and may occur even at low-dose exposures [4]. PFOA and PFOS are both found in human breast milk [8, 12, 13]. Consistent inverse associations between circulating PFAS concentrations and breastfeeding duration [14, 15] may indicate deleterious effects of PFAS on normal breast development and function, in agreement with experimental findings. Prior epidemiologic studies offer preliminary evidence that exposure to certain PFAS may be associated with a doubling of breast cancer risk [16–18], although null associations have been reported by other studies [17, 19, 20]. Thus, whether PFAS exposure contributes to human breast cancer risk remains unclear.

Exploring features of human breast tissue can provide important insights on breast carcinogenesis and the impacts of specific exposures. Terminal ductal lobular units (TDLUs) are the structures that produce milk following childbirth, and involution of TDLUs normally occurs following lactation and with aging and the menopausal transition. Most breast cancers arise from TDLUs, and a greater degree of TDLU involution (e.g., fewer TDLUs) has been associated with lower breast cancer risk in multiple studies [21–24], though not all [25].

To explore potential associations between PFAS concentrations and breast carcinogenesis, we leveraged prior measurements of TDLU involution from breast tissue samples provided by cancer-free volunteer participants from the Susan G. Komen for the Cure Tissue Bank (KTB).

## Methods

### Study population

The Susan G. Komen for the Cure Tissue Bank (KTB) at the Indiana University Simon Cancer Center was established in 2007 to document the molecular histology of the normal breast [26]. Volunteer participants provided epidemiologic data and blood specimens and underwent a breast biopsy to obtain breast tissue samples. To be eligible to donate tissue to the KTB, donors met the following criteria: (1) biological female, (2) age ≥ 18 year, (3) no breast implants, (4) no known allergy to local anesthetics, (5) not currently taking a blood thinner except aspirin, and (6) no prior chest radiation. Data on KTB participants are publicly available to researchers through the Virtual Tissue Bank (https://virtualtissuebank.iu.edu).

We identified KTB participants who met the following eligibility criteria: (1) no history of breast cancer or ductal carcinoma in situ, (2) postmenopausal, (3) no current hormone therapy (HT) use, (4) available serum sample, (5) available postmenopausal mammogram(s), and (6) available postmenopausal TDLU involution measurements. We further excluded participants who were subsequently diagnosed with breast cancer (*n* = 14), who were identified to have a rare genetic mutation related to breast cancer risk (*n* = 4), or who had an unusable quality serum specimen (*n* = 1), as these samples were not provided by the KTB. The present analysis included 263 participants.

All KTB participants provided written informed consent at the time of donation. Human subjects approval for the KTB was provided by the Institutional Review Board at Indiana University. The present research utilized fully deidentified data and previously collected biospecimens and thus was determined to not qualify as human subjects research by the Institutional Review Board at the University of Massachusetts Amherst.

### PFAS measurement

Briefly, at the time of breast biopsy serum samples were obtained from blood drawn into 9mL collection tubes and immediately centrifuged for 15 min at 2000 rcf. The serum layer was then transferred into separate 750 µL aliquots in cryogenic vials and stored at -80 °C. 750 uL serum samples were retrieved for selected participants and shipped to Clinical Pharmacology, Pharmacy and Environmental Medicine, University of Southern Denmark for PFAS analysis packed in dry ice via overnight courier. All laboratory staff were masked to participants’ characteristics.

The laboratory measured a panel of seven PFAS using online solid-phase extraction followed by high-pressure liquid chromatography with tandem mass spectrometry [27, 28]: perfluorooctanoate (PFOA), perfluorooctanoic sulfonate (PFOS), perfluorohexanoic sulfonate (PFHxS), perfluoroheptane sulfonate (PFHpS), perfluorononaoate (PFNA), perfluorodecanoate (PFDA), and perfluoroundecanoate (PFUnDA). The limit of detection (LOD) was 0.03 ng/ml for all PFAS; values < LOD were set to 0.015 ng/mL for analysis (0% PFOA, 1% PFHxS, 0% PFNA, 5% PFHpS, 0% PFDA, 0% PFOS, 6% PFUnDA). Coefficients of variation (CV) were calculated for each PFAS: PFOA (4.1%), PFOS (9.1%), PFNA (4.7%), PFDA (6.3%), PFHxS (10.1%), PFHpS (13.7%), PFUnDA (5.5%). Samples were randomly distributed through batches. No significant variation across batches was observed (for each PFAS, *p* > 0.9 ANOVA testing batch effect in quality control samples). The accuracy of the analytical method for PFAS was ensured by participation in the German External Quality Assessment Scheme (G-EQUAS), organized by Social and Environmental Medicine of the University of Erlangen-Nuremberg.

### TDLU measurements

KTB volunteers provided up to six core samples from the upper outer quadrant of a single breast. Needle biopsies were performed under local anesthetic by a surgeon using a 10-gauge needle. Breast tissue samples were immediately processed following biopsy. One core sample was fixed in 10% buffered formalin and processed as paraffin-embedded blocks, from which a 5 μm section was obtained and stained with hematoxylin and eosin (H&E). Digital images of H&E stained tissues are available in the Virtual Tissue Bank.

Prior work assessed three measures of TDLU involution using the digitized H&E sections: TDLU counts per standardized biopsy, median acini counts per TDLU, and median TDLU span [29, 30]. Briefly, a trained pathologist assessed up to ten normal TDLUs to measure: (a) the TDLU span, using an electronic ruler to measure in microns, and (b) the number of acini per TDLU. The median values for each of these measures across the TDLUs assessed were used in analyses. A section of breast tissue (median 34.5 mm^2^) was inspected and the number of TDLUs observed within that section was recorded as the TDLU count.

We utilized three parametrizations of TDLU involution for analysis as outcomes of interest. First, we created a dichotomous variable indicating any observed TDLUs (no, yes). Second, a variable indicating the number of observed TDLUs was created, with values of 0 for participants without observed TDLUs (integers). Third, among participants with observed TDLUs, we calculated the median TDLU span as the median diameter among the measured TDLUs (continuous). Each of these quantitative measurements is negatively associated with TDLU involution (i.e., *higher* TDLU count, TDLU span, and median number of acini per TDLU demonstrate *less* involution). These measurements were assessed for both intra- and inter-observer agreement and were demonstrated to be highly reliable and highly correlated with qualitative assessments of TDLU involution [29].

### Covariate data

Additional data were obtained from questionnaires completed by KTB participants at the time of sample donation. Participants self-reported their age, education, race, history of tobacco use, history of pregnancies and live births, breastfeeding history, ages at menarche and menopause, and history of postmenopausal hormone therapy (HT) use. Additionally, body mass index (BMI) was calculated from height and weight measured by a research nurse at the enrollment visit. Lifetime risk Gail score [31] was calculated using self-reported data collected at the time of enrollment.

### Statistical analysis

We calculated age-adjusted descriptive statistics for all covariates, stratified by any observed TDLUs (no, yes). We calculated geometric means for the measured PFAS and tested for differences between those with and without any observed TDLUs using t tests. SAS (version 9.4) was used for these analyses.

Based on prior knowledge and causal diagrams, we included the following variables as covariates in our analysis: age (continuous), race (White, non-White), lifetime risk Gail score (continuous), BMI (continuous), ever used tobacco (no, yes), age at menarche (continuous), ever pregnant (no, yes), time since last menstrual period (continuous), ever used postmenopausal HT (no, yes), and percent fat viewed in the tissue Sects. (0–25%, 26–50%, 51–75%, and 76–100%).

We used Bayesian Kernel Machine Regression (BKMR) to statistically explore the individual and joint effects of the chemical mixture of PFAS, using the R package “bkmr”. BKMR is a semi-parametric, supervised approach for the detection of the toxic agent(s) that uses a Bayesian approach to variable selection within a kernel machine regression [32]. BKMR allows the visualization of the exposure-response association for each mixture component, while accounting for correlation between mixture components, and it also estimates the multivariable exposure-response function in a flexible way that allows for nonlinear and non-additive effects. Prior to fitting BKMR models, each PFAS measure was first natural log-transformed and then standardized by subtracting its mean and dividing by its standard deviation. We used Quantile-G computation to estimate the joint mixture effect, using the R package “qgcomp.” Adjusting for the aforementioned covariates, the individual positive and negative effects of each PFAS and its contribution to the overall mixture effect were calculated.

We repeated the above analyses [1] restricted to the subgroup of women who had never used postmenopausal HT, and [2] among parous women with stratification on breastfeeding history; these models were not adjusted for percent fat in the viewed tissue section due to numerical instability given the small numbers in stratified analyses and that the vast majority of samples had 76–100% fat.

We performed BKMR and Quantile-G computation analyses on the following outcomes: observed TDLUs (none, any), number of TDLUs observed (integers), number of TDLUs observed among those with > 0 observed TDLUs (integers), and median TDLU span among those with any observed TDLUs (continuous). BKMR and Quantile g-computation analyses were performed using R 4.4.2. Additionally, because BKMR does not allow for incorporation of zero-inflated regression, we ran exploratory analyses using negative binomial regression with TDLU count as the outcome and individual PFAS as the exposures using SAS version 9.4 (SAS Institute, Cary, NC).

## Results

The average age of participants was 58 years among both those participants with and without observed TDLUs (Table 1). Participants with observed TDLUs (i.e., less involution of breast tissue) were less frequently of White race (63.1% vs. 75.5%), more frequently reported a history of pregnancy (87.9% vs. 77.4%), and had a slightly longer time since last menstrual period (13.5 vs. 12.3 years).

**Fig. 1:**
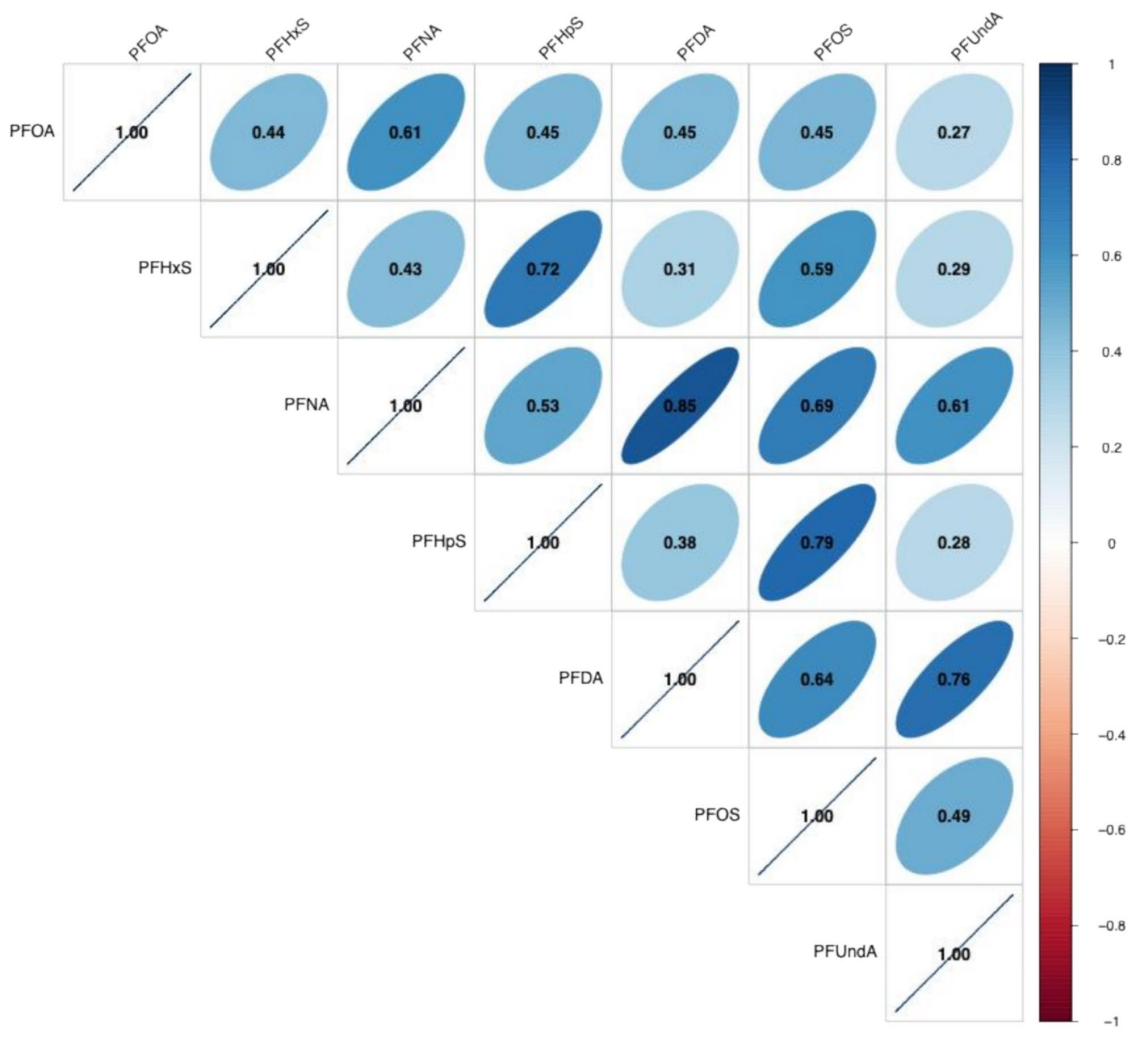
Pairwise correlations between measured PFAS (*N* = 263)

Overall, distributions of PFAS were similar between participants with and without observed TDLUs (Table 2). We observed positive correlations between all measured PFAS (Fig. 1). In particular, several strong correlations (*r* > 0.6) were observed: PFNA and PFOA (*r* = 0.61), PFHpS and PFHxS (*r* = 0.72), PFDA and PFNA (*r* = 0.85), PFOS and PFNA (*r* = 0.70), PFUndA and PFNA (*r* = 0.61), PFOS and PFHpS (*r* = 0.79), PFOS and PFDA (*r* = 0.64), PFUndA and PFDA (*r* = 0.76).

**Table 1:** Descriptive characteristics of study population by TDLU status (*N* = 263)

	Any TDLUs observed		
	No (*N* = 106 [40.3%])	Yes (*N* = 157 [59.7%])	*P* value
Age, years; Mean (SD)	58.6 (8.1)	58.7 (7.6)	0.97
Age, years; Min – Max	40–76	33–75	
Education; N (%)			0.11
High School or Less	16 (15.1)	43 (27.4)	
Associate’s/Bachelor’s Degree	37 (34.9)	52 (33.1)	
Graduate/Professional Degree	39 (36.8)	47 (29.9)	
Other/Unknown	14 (13.2)	15 (9.6)	
White race; N (%)	80 (75.5)	99 (63.1)	0.03
Gail Score (lifetime risk); Mean (SD)	10.3 (5.0)	10.0 (5.5)	0.70
Body mass index, kg/m^2^; Mean (SD)	30.6 (7.1)	29.3 (6.7)	0.13
Ever used tobacco; N (%)	33 (31.1)	47 (30.0)	0.84
Age at menarche, years; Mean (SD)	12.7 (1.4)	12.8 (1.5)	0.80
Ever pregnant; N (%)	82 (77.4)	138 (87.9)	0.02
Number live births;^a^ N (%)	2.2 (1.2)	2.2 (1.2)	0.94
Ever breastfed^a^; N (%)	51 (63.8)	87 (64.0)	0.97
Time since last menstrual period, yrs; Mean (SD)	12.3 (10.0)	13.5 (10.4)	0.35
Ever used postmenopausal HT; N (%)	38 (35.9)	49 (31.2)	0.43
Amount of fat present in examined tissue; N (%)			< 0.001
0–25%	0 (0)	6 (3.8)	
26–50%	2 (1.9)	15 (9.6)	
51–75%	0 (0.0)	32 (20.4)	
76–100%	104 (98.1)	104 (66.2)	

^a^ Among those ever pregnant

**Table 2:** Summary of estimates of overall mixture effect and individual contribution to the overall mixture effect for each outcome using Quantile-G computation (*N* = 263)^a^

	TDLUs Observed				
	No (*N* = 106)		Yes (*N* = 157)		
PFAS Chemical	Geometric Mean (SD)	Min - Max	Geometric Mean (SD)	Min - Max	*P* value
PFOA, ng/mL	2.64 (2.20)	0.17–20.84	2.52 (1.72)	0.41–9.95	0.57
PFHxS, ng/mL	0.90 (2.76)	0.02–7.19	1.12 (2.30)	0.02–7.74	0.06
PFNA, ng/mL	0.76 (1.62)	0.16–2.62	0.83 (1.69)	0.10–4.47	0.22
PFHpS, ng/mL	0.15 (2.81)	0.02–0.97	0.18 (2.43)	0.02–1.32	0.12
PFOS, ng/mL	6.99 (2.15)	0.86–102.83	7.68 (2.04)	0.23–42.36	0.31
PFUnDA, ng/mL	0.10 (2.29)	0.02–0.53	0.10 (2.35)	0.02–1.07	0.95
PFDA, ng/mL	0.22 (1.67)	0.07–0.98	0.23 (1.88)	0.02–1.57	0.37

BKMR analysis was used to explore the independent and combined effects of the PFAS mixture components (PFOA, PFDA, PFUnDA, PFHpS, PFHxS, PFNA, PFOS) on the outcome of any observed TDLUs (yes vs. no) (Supplemental Fig. 1). Adjusting for other mixture components and covariates, higher concentrations of PFOA increased the odds of any observed TDLUs until approximately the median, after which higher concentrations of PFOA were associated with lower odds of observed TDLUs. PFHxS, PFNA, and PFHpS demonstrated positive associations between concentrations of these chemicals and odds of observed TDLUs. PFUnDA demonstrated a negative association with odds of observed TDLUs. No qualitative associations were observed between PFDA or PFOS and odds of observed TDLUs.

Overall, similar trends were observed when evaluating the effect of each component per one SD increase when setting the other mixture components at specific quantiles, although none were identified as statistically significant (Supplemental Fig. 2). In particular, negative associations were observed for PFUnDA and odds of observed TDLUs at the 25th, 50th, and 75th quantiles of the mixture. Suggested positive associations with odds of observed TDLUs were apparent for PFDA at the 75th quantile of the mixture and for PFHpS and PFHxS at the 25th quantile of the mixture. A negative association between PFOA and odds of observed TDLUs was suggested at the 25th quantile of the mixture. Together, the overall effect of the mixture, adjusting for covariates, suggested an inverted U-shape association between the PFAS mixture and odds of observed TDLUs, whereby both lower mixture concentrations and higher mixture concentrations were negatively associated with odds of observed TDLUs, although these associations did not achieve statistical significance (Supplemental Fig. 3). When exploring bivariate interactions, we observed apparent interactions between concentrations of PFUnDA and other PFAS. These plots suggested that the effect of each other mixture component on odds of observed TDLUs was impacted by the concentration of PFUnDA (Supplemental Fig. 4).

Next, we repeated analyses with the number of observed TDLUs as the outcome (Supplemental Figs. 5–8). Overall, there were no apparent associations between individual PFAS mixture components and number of TDLUs. For the overall mixture effect there was some suggestion of a negative association with number of observed TDLUs, indicating greater involution, although this was not statistically significant. These results were confirmed by the exploratory analyses using negative binomial regression (data not shown).

Because nearly half of the participants had no observed TDLUs (*n* = 106), we performed further analyses restricted to those participants with observed TDLUs (*n* = 157). No associations were observed between individual mixture components or the overall mixture and the number of observed TDLUs (data not shown). Among this subgroup, there was some suggestion that PFOS concentrations were negatively associated with the number of TDLUs when the other mixture components were at the 25th percentile, indicating greater involution. There were no apparent associations between the individual mixture components or the overall mixture and median diameter of acini in the observed TDLUs (data not shown).

The above analyses were repeated among the subgroup of participants who did not use postmenopausal HT; results were similar to those observed within the full study population for all outcomes (data not shown).

**Fig. 2:**
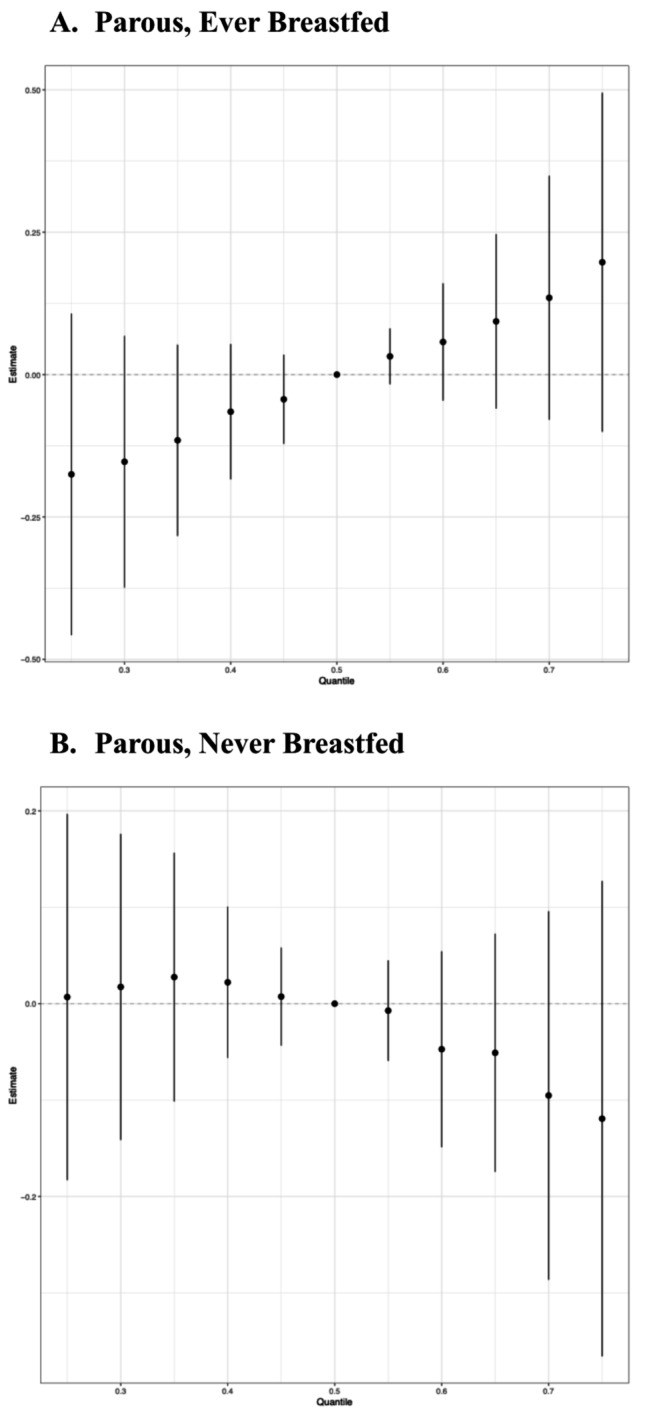
Plots of the overall mixture effect on the outcome of any vs. none observed TDLUs, separately **(A)** among parous women who ever breastfed (*N* = 138) and **(B)** among parous women who had never breastfed (*N* = 78) ^a^. ^a^ Adjusted for age, race, body mass index, lifetime Gail risk score, age at menarche, ever been pregnant, years since menopause, ever use of postmenopausal HT

**Fig. 3:**
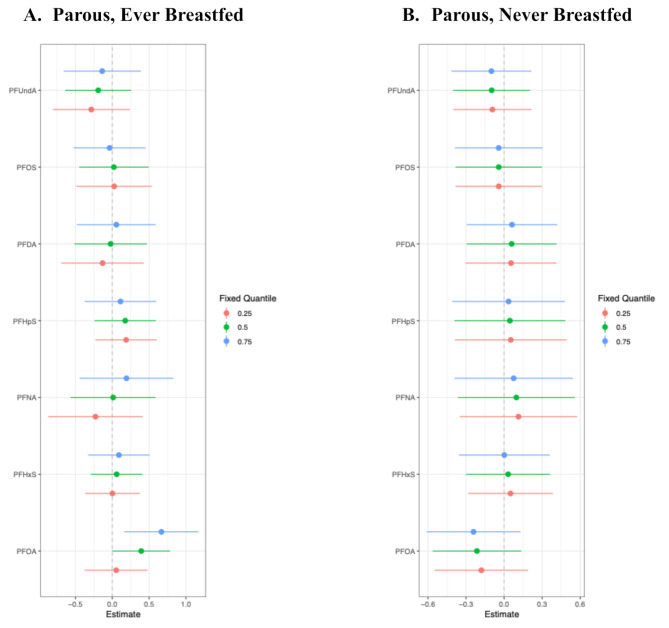
Effects of each PFAS chemical when other mixture components are held at the 25th, 50th, or 75th percentiles, separately among parous women who **(A)** ever (*N* = 178) or **(B)** never (*N* = 78) breastfed ^a^. ^a^ Adjusted for age, race, body mass index, lifetime Gail risk score, age at menarche, ever been pregnant, years since menopause, ever use of postmenopausal HT

Next, we explored associations between PFAS and TDLU involution among parous women, with stratification on breastfeeding history. Interestingly, the BKMR analysis identified a positive association between the overall mixture effect and observed TDLUs among parous women who had ever breastfed, indicating less involution (Fig. 2A), but a slightly inverse association among parous women who had never breastfed, indicating greater involution (Fig. 2B). The difference appeared to be driven by differential associations with PFOA when other mixture components were at the 75th percentile; in these analyses, there was a positive association between PFOA and any observed TDLUs (yes vs. no) among parous women who had ever breastfeed (Fig. 3A), yet a negative association between PFOA and observed TDLUs among parous women who had never breastfed (Fig. 3B).

Among the full study population, the results of the Quantile-G computation analysis indicated no overall effect of the PFAS mixture for any of the outcomes we evaluated (Table 3). PFHpS had a consistently positive direction of effect across outcomes, while both PFUnDA and PFDA had consistently negative effects. The direction of effect for the remaining PFAS evaluated (PFOA, PFHxS, PFNA) varied across the four analyses. Similar results were observed among the subgroups of participants who did not use postmenopausal HT and when stratified by breastfeeding history among parous women (data not shown).

**Table 3:** Summary of estimates of overall mixture effect and individual contribution to the overall mixture effect for each outcome using Quantile-G computation (*N*=263)^a^

	Any vs. None TDLU	Number TDLU	Number TDLU^b^	Median TDLU Span^b^
***Overall Mixture Effect*** ***Beta (95% CI);*** *p*				
	0.03 (-2.75, 2.81); *p* = 0.98	-0.15 (-1.04, 0.73); *p* = 0.74	-0.59 (-1.90, 0.72); *p* = 0.38	-4.59 (-22.97, 13.79); *p* = 0.63
	***Individual Weights and Direction of Effect***			
PFOA	-0.010	0.27	0.23	-0.30
PFHxS	0.32	0.13	0.04	-0.35
PFNA	0.18	-0.08	-0.37	0.33
PFHpS	0.26	0.44	0.25	0.44
PFOS	0.24	-0.38	-0.37	0.23
PFUnDA	-0.89	-0.54	-0.27	-0.09
PFDA	-0.10	0.15	0.49	-0.26

^a^ Adjusted for age, race, body mass index, lifetime Gail risk score, age at menarche, ever been pregnant, years since menopause, ever use of postmenopausal HT, percent fat in tissue section

^b^ Among those with observed TDLUs

## Discussion

Overall, we observed no meaningful associations between serum PFAS concentrations and TDLU involution in this sample of postmenopausal women. Among parous women, stratified analyses using BKMR indicated that the mixture effect may differ by breastfeeding history. These analyses suggested a positive association between PFAS and the presence of TDLUs among those with a history of breastfeeding, yet a negative association among those who had never breastfed. However, the quantile G-computation analysis did not support differential effects by breastfeeding history. The number of nulliparous women were too small to support separate analyses within this subgroup.

This is, to our knowledge, the first study to evaluate potential associations between PFAS and TDLU involution. Recently, higher PM2.5 levels were associated with higher TDLU counts, indicating that greater exposure to this common air pollutant parameter was associated with lower breast tissue involution [33]. Such results signal that environmental exposures may have important impacts on breast tissue remodeling and, ultimately, breast cancer risk.

Several epidemiologic studies have explored associations between PFAS and breast cancer risk. A recent meta-analysis of prospective studies [34] estimated a slight, yet non-significant, increase in risk associated with PFOA (4 studies; RR = 1.16, 95% CI 0.96–1.40), but no association with PFOS (4 studies; RR = 1.03, 95% CI 0.87–1.22), PFHxS (2 studies; RR = 0.79, 95% CI 0.51–1.23), or PFNA (2 studies; RR = 1.17, 95% CI 0.86–1.59). While few studies included in the meta-analysis explored associations between PFAS and hormone-receptor subtypes among postmenopausal women, a possible positive association between PFOA and ER- tumors was noted (RR = 1.55, 95% CI 0.90–2.67), with no association between PFOA and ER + cancers (RR = 0.97, 95% CI 0.67–1.39). Individual studies provided heterogeneous results when evaluating associations between PFOS and ER+/- cancers, with overall estimates null (ER+: RR = 1.07, 95% CI 0.62–1.84; ER- RR = 0.90, 95% CI 0.56–1.42) [34]. Given that many risk factors, including breast feeding, differentially impact breast cancer risk, the possibility that PFOA may increase risk of ER-, but not ER+, breast cancer is intriguing. However, the relative lack of prospective studies precludes any definitive conclusions. Future research using prospective data, measuring a broad panel of PFAS, and separately examining hormone-receptor subtypes is needed.

Animal studies provide substantial evidence that PFAS affect breast tissue morphology. PFOA exposure delays mammary gland development in mouse models [2]. Separate studies showed that these effects persist across three generations [3, 5] and also result from low-dose exposures [4]. Additionally, pregnant mice exposed to PFOA displayed stunted branching of the mammary epithelium and delayed involution [1]. Consistent with these deleterious effects on mammary gland development and function, epidemiological studies have reported significant inverse associations between circulating PFAS concentrations and breastfeeding duration [14, 15]. Our finding of differential associations among parous women by breastfeeding history may be consistent with this observation. TDLUs produce milk during lactation. If PFAS exposure results in fewer TDLUs, and thus less breastfeeding, then this may appear as greater involution (i.e., lower or no TDLU counts) among those who never breastfed, although their TDLU count initially would have been lower compared to breastfeeding women. Change in TDLU count could add important insight, although we note that prospective repeated measures of TDLU involution are not available and would be extremely challenging to collect in a cancer-free volunteer population.

The majority of breast cancers arise from TDLUs. Breast tissue changes throughout the life course, and low TDLU involution may foreshadow a future breast cancer diagnosis. Greater TDLU involution in women with benign breast disease (BBD) has been associated with lower breast cancer risk in several studies [21–24], though not all [25]. For example, a case-control study of women with BBD observed a non-significant two-fold higher risk among women with the highest TDLU counts compared to those with the lowest (4th vs. 1st quartile, OR = 2.44, 95% CI 0.96–6.19) [24]. However, prior work has not evaluated associations between TDLU involution measures and future breast cancer risk using breast tissue samples from cancer-free volunteers, similar to the KTB participants studied in the present research. Therefore, the lack of association we observed between PFAS and TDLU involution does not definitively rule out an effect of PFAS on future breast cancer risk. Additionally, TDLUs naturally involute with chronological aging. The lack of association we observed may be due to the older, postmenopausal age of our study population. Future research should assess whether PFAS are associated with TDLU involution in premenopausal women.

Also, while *greater* TDLU involution is associated with *lower* breast cancer risk in women with BBD, prior analyses within the KTB indicate that some factors known to increase breast cancer risk are also associated with greater TDLU involution [29]. For example, nulliparous women had lower TDLU counts than women with one live birth (RR = 0.67, 95% CI 0.56–0.97), and increased parity also was positively associated with TDLU counts (RR = 1.20, 95% CI 1.04–1.37), despite parity having a well-established negative association with future breast cancer risk. A similar paradoxical association was observed with breastfeeding history, with the risk of having observed TDLUs estimated to be 24% higher among postmenopausal women with a history of breastfeeding compared to postmenopausal women who had never breastfed (RR = 1.24, 95% CI 1.11–1.38) [29]. This finding is consistent with the idea that breastfeeding is associated with more TDLUs, and thus TDLU counts in postmenopausal women may distinguish extent of involution across women only when first stratified by breastfeeding history.

Our findings must be considered in the context of several limitations. KTB donors are majority White and residents of the U.S. state of Indiana. While 32% of our study population was non-White, future research in more racially and ethnically diverse populations is needed. Also, our sample population was limited to postmenopausal women not currently using HT; it is possible that different results would be observed among premenopausal women and among postmenopausal women currently taking HT medications. We acknowledge that we measured PFAS exposure at a single point in time that was coincident with the breast biopsy. However, serum PFAS measurements are accepted as reflecting long-term (~ 5–10 years prior) exposure, thus supporting that measured concentrations of PFAS reflect exposures that preceded the measurements of TDLU involution. Further, while we were specifically powered to test our hypotheses among the full sample population, results of stratified analyses should be interpreted cautiously given the smaller sample sizes and resulting limited statistical power. Also, the BKMR analysis that was our primary analytic approach did not support modeling TDLU counts, thus we were limited to analyzing these data as any TDLUs observed (yes vs. no). We ran exploratory analyses using negative binomial regression with TDLU count as the outcome and individual PFAS as the exposures, which confirmed the null associations resulting from the BKMR analyses. Finally, the TDLU measurements arose from examination of a single section from a core biopsy and may not be representative of the entire breast. Non-differential misclassification may therefore have impacted our analysis, possibly biasing results towards the null. However, prior work has established the validity of a single sample to reflect the extent of involution in the breast [35, 36], and we thus anticipate such effects to be minimal.

Our analysis was strengthened by many unique methodological features. First, we utilized breast tissue samples donated from a volunteer population of cancer-free women. Most explorations involving histologic breast tissue measures utilize tissue from breast cancer cases, women undergoing prophylactic mastectomy, or women undergoing reduction mammoplasty. Thus, our findings have potentially greater generalizability to cancer-free women. Additionally, we measured a broad panel of PFAS, including those with the highest prevalence of exposure in the U.S. population. Because each PFAS may have different effects, it is important to consider compounds beyond PFOA and PFOS, which have received most of the attention in epidemiologic studies. Finally, we utilized statistical analysis approaches that facilitate modeling concentrations of PFAS as a chemical mixture, more closely reflecting human exposures. These analyses allowed us to evaluate both individual effects of each PFAS in the context of exposure to the other PFAS in addition to the effect of the PFAS mixture itself.

## Conclusions

Overall, our analysis does not support meaningful effects of PFAS on TDLU involution, although we note that these findings are not applicable to premenopausal women or to postmenopausal women using HT. Also, it is possible that PFAS promote breast carcinogenesis via a mechanism that does not include effects on TDLU involution. Therefore, the lack of observed association between PFAS and TDLU involution in our analyses does not fully rule out an impact of PFAS on breast cancer risk.

.

## Supplementary Information

Below is the link to the electronic supplementary material.

Supplementary Material 1

## Data Availability

Data on Komen Tissue Bank participants are publicly available to researchers through the Virtual Tissue Bank (https://virtualtissuebank.iu.edu).

## Abbreviations

BKMR: Bayesian kernel machine regression
BMI: Body mass index
HT: Hormone therapy
KTB: Komen Tissue Bank
LOD: Limit of detection
PFAS: Per- and polyfluoroalkyl substance
PFDA: Perfluorodecanoate
PFHpS: Perfluoroheptane sulfonate
PFHxS: Perfluorohexanoic sulfonate
PFNA: Perfluorononaoate
PFOA: Perfluoroocatanoic acid
PFOS: Perfluorooctane sulfate
PFUnDA: Perfluoroundecanoate
TDLU: Terminal ductal lobular unit

## References

[1] White SS, Calafat AM, Kuklenyik Z, Villanueva L, Zehr RD, Helfant L, et al. Gestational PFOA exposure of mice is associated with altered mammary gland development in dams and female offspring. Toxicol Sci Off J Soc Toxicol. 2007;96(1):133–44. 10.1093/toxsci/kfl177 17132714

[2] White SS, Kato K, Jia LT, Basden BJ, Calafat AM, Hines EP, et al. Effects of perfluorooctanoic acid on mouse mammary gland development and differentiation resulting from cross-foster and restricted gestational exposures. Reprod Toxicol Elmsford N. 2009 ;27(3–4):289–98. 10.1016/j.reprotox.2008.11.054 PMC3477546 19095057

[3] White SS, Stanko JP, Kato K, Calafat AM, Hines EP, Fenton SE. Gestational and chronic Low-Dose PFOA exposures and mammary gland growth and differentiation in three generations of CD-1 mice. Environ Health Perspect. 2011;119(8):1070–6. 21501981 10.1289/ehp.1002741 PMC3237341

[4] Macon MB, Villanueva LR, Tatum-Gibbs K, Zehr RD, Strynar MJ, Stanko JP, et al. Prenatal perfluorooctanoic acid exposure in CD-1 mice: low-dose developmental effects and internal dosimetry. Toxicol Sci Off J Soc Toxicol. 2011;122(1):134–45. 10.1093/toxsci/kfr076 PMC3143465 21482639

[5] Tucker DK, Macon MB, Strynar MJ, Dagnino S, Andersen E, Fenton SE. The mammary gland is a sensitive pubertal target in CD-1 and C57Bl/6 mice following perinatal perfluorooctanoic acid (PFOA) exposure. Reprod toxicol Elmsford N. 2015 ;54:26–36. 10.1016/j.reprotox.2014.12.002 PMC4464984 25499722

[6] IARC. Perfluorooctanoic Acid (PFOA). and Perfluorooctanesulfonic Acid (PFOS) [Internet]. [cited 2025 May 28]. Available from: https://publications.iarc.who.int/Book-And-Report-Series/Iarc-Monographs-On-The-Identification-Of-Carcinogenic-Hazards-To-Humans/Perfluorooctanoic-Acid-PFOA-And-Perfluorooctanesulfonic-Acid-PFOS--2025 40489584

[7] Calafat AM, Wong LY, Kuklenyik Z, Reidy JA, Needham LL. Polyfluoroalkyl chemicals in the U.S. Population: data from the National health and nutrition examination survey (NHANES) 2003–2004 and comparisons with NHANES 1999–2000. Environ Health Perspect. 2007;115(11):1596–602. 18007991 10.1289/ehp.10598 PMC2072821

[8] Mogensen UB, Grandjean P, Nielsen F, Weihe P, Budtz-Jørgensen E. Breastfeeding as an exposure pathway for perfluorinated alkylates. Environ Sci Technol. 2015 ;49(1):10466–73. 10.1021/acs.est.5b02237 PMC6190571 26291735

[9] Pérez F, Nadal M, Navarro-Ortega A, Fàbrega F, Domingo JL, Barceló D, et al. Accumulation of perfluoroalkyl substances in human tissues. Environ Int. 2013 ;59:354–62. 10.1016/j.envint.2013.06.004 23892228

[10] Li Y, Fletcher T, Mucs D, Scott K, Lindh CH, Tallving P, et al. Half-lives of PFOS, PFHxS and PFOA after end of exposure to contaminated drinking water. Occup Environ Med. 2018;75(1):46–51. 29133598 10.1136/oemed-2017-104651 PMC5749314

[11] International Agency for Research on Cancer (IARC). Perfluorooctanoic Acid, Tetrafluoroethylene, Dichloromethane, 1,2-Dichloropropane, and 1,3-Propane Sultone [Internet]. 2016. (IARC Monographs Volume 110). Available from: https://publications.iarc.fr/547

[12] LaKind JS, Naiman J, Verner MA, Lévêque L, Fenton S. Per- and polyfluoroalkyl substances (PFAS) in breast milk and infant formula: A global issue. Environ Res. 2023;219:115042. 36529330 10.1016/j.envres.2022.115042 PMC9872587

[13] LaKind JS, Verner MA, Rogers RD, Goeden H, Naiman DQ, Marchitti SA, et al. Current breast milk PFAS levels in the united States and canada: after all this time, why don’t we know more?? Environ Health Perspect. 2022;130(2):25002. 35195447 10.1289/EHP10359 PMC8865090

[14] Romano ME, Xu Y, Calafat AM, Yolton K, Chen A, Webster GM, et al. Maternal serum perfluoroalkyl substances during pregnancy and duration of breastfeeding. Environ Res. 2016;149:239–46. 27179585 10.1016/j.envres.2016.04.034 PMC4907828

[15] Timmermann CAG, Budtz-Jørgensen E, Petersen MS, Weihe P, Steuerwald U, Nielsen F, et al. Shorter duration of breastfeeding at elevated exposures to perfluoroalkyl substances. Reprod Toxicol Elmsford N. 2017;68:164–70. 10.1016/j.reprotox.2016.07.010 PMC5233673 27421579

[16] Mancini FR, Cano-Sancho G, Gambaretti J, Marchand P, Boutron-Ruault MC, Severi G, et al. Perfluorinated alkylated substances serum concentration and breast cancer risk: evidence from a nested case-control study in the French E3N cohort. Int J Cancer. 2020;146(4):917–28. 31008526 10.1002/ijc.32357

[17] Tsai MS, Chang SH, Kuo WH, Kuo CH, Li SY, Wang MY, et al. A case-control study of perfluoroalkyl substances and the risk of breast cancer in Taiwanese women. Environ Int. 2020;142:105850. 10.1016/j.envint.2020.105850 32580117

[18] Wielsøe M, Kern P, Bonefeld-Jørgensen EC. Serum levels of environmental pollutants is a risk factor for breast cancer in inuit: a case control study. Environ Health Glob Access Sci Source. 2017;13(1):56. 10.1186/s12940-017-0269-6 PMC5470290 28610584

[19] Bonefeld-Jørgensen EC, Long M, Fredslund SO, Bossi R, Olsen J. Breast cancer risk after exposure to perfluorinated compounds in Danish women: a case-control study nested in the Danish National birth cohort. Cancer Causes Control CCC. 2014;25(11):1439–48. 25148915 10.1007/s10552-014-0446-7 PMC4215104

[20] Velarde MC, Chan AFO, Sajo MEJV, Zakharevich I, Melamed J, Uy GLB, et al. Elevated levels of perfluoroalkyl substances in breast cancer patients within the greater Manila area. Chemosphere. 2022;286(Pt 1):131545. 34293563 10.1016/j.chemosphere.2021.131545

[21] Milanese TR, Hartmann LC, Sellers TA, Frost MH, Vierkant RA, Maloney SD, et al. Age-related lobular Involution and risk of breast cancer. J Natl Cancer Inst. 2006;98(22):1600–7. 17105983 10.1093/jnci/djj439

[22] Baer HJ, Collins LC, Connolly JL, Colditz GA, Schnitt SJ, Tamimi RM. Lobule type and subsequent breast cancer risk: results from the nurses’ health studies. Cancer. 2009;115(7):1404–11. 19170235 10.1002/cncr.24167 PMC2661011

[23] McKian KP, Reynolds CA, Visscher DW, Nassar A, Radisky DC, Vierkant RA, et al. Novel breast tissue feature strongly associated with risk of breast cancer. J Clin Oncol. 2009;27(35):5893–8. 19805686 10.1200/JCO.2008.21.5079 PMC2793038

[24] Figueroa JD, Pfeiffer RM, Brinton LA, Palakal MM, Degnim AC, Radisky D, et al. Standardized measures of lobular Involution and subsequent breast cancer risk among women with benign breast disease: a nested case-control study. Breast Cancer Res Treat. 2016;159(1):163–72. 27488681 10.1007/s10549-016-3908-7 PMC5045857

[25] Kensler KH, Liu EZF, Wetstein SC, Onken AM, Luffman CI, Baker GM, et al. Automated quantitative measures of terminal duct lobular unit Involution and breast cancer risk. Cancer Epidemiol Prev Biomark. 2020;29(11):2358–68. 10.1158/1055-9965.EPI-20-0723 PMC7642012 32917665

[26] Sherman ME, Figueroa JD, Henry JE, Clare SE, Rufenbarger C, Storniolo AM, The Susan G. Komen for the cure tissue bank at the IU Simon cancer center: A unique resource for defining the molecular histology of the breast. Cancer Prev Res (Phila Pa). 2012;5(4):528–35. 10.1158/1940-6207.CAPR-11-0234 PMC4159749 22345117

[27] Haug LS, Thomsen C, Becher G. A sensitive method for determination of a broad range of perfluorinated compounds in serum suitable for large-scale human biomonitoring. J Chromatogr A. 2009;1216(3):385–93. 19026423 10.1016/j.chroma.2008.10.113

[28] Nielsen F, Fischer FC, Leth PM, Grandjean P. Occurrence of major perfluorinated alkylate substances in human blood and target organs. Environ Sci Technol. 2024;58(1):143–9. 38154793 10.1021/acs.est.3c06499 PMC10785751

[29] Figueroa JD, Pfeiffer RM, Patel DA, Linville L, Brinton LA, Gierach GL et al. Terminal duct lobular unit Involution of the normal breast: implications for breast cancer etiology. J Natl Cancer Inst. 2014;106(10): 1-11 . 10.1093/jnci/dju286 PMC4200067 25274491

[30] Khodr ZG, Sherman ME, Pfeiffer RM, Gierach GL, Brinton LA, Falk RT, et al. Circulating sex hormones and terminal duct lobular unit Involution of the normal breast. Cancer Epidemiol Biomark Prev Publ Am Assoc Cancer Res Cosponsored Am Soc Prev Oncol. 2014;23(12):2765–73. 10.1158/1055-9965.EPI-14-0667 PMC4333643 25472681

[31] Gail MH, Brinton LA, Byar DP, Corle DK, Green SB, Schairer C, et al. Projecting individualized probabilities of developing breast cancer for white females who are being examined annually. J Natl Cancer Inst. 1989;81(24):1879–86. 2593165 10.1093/jnci/81.24.1879

[32] Bobb JF, Valeri L, Claus Henn B, Christiani DC, Wright RO, Mazumdar M et al. Bayesian kernel machine regression for estimating the health effects of multi-pollutant mixtures. Biostatistics. 2015 July 1;16(3):493–508. 10.1093/biostatistics/kxu058 PMC5963470 25532525

[33] Niehoff NM, Keil AP, Jones RR, Fan S, Gierach GL, White AJ. Outdoor air pollution and terminal duct lobular Involution of the normal breast. Breast Cancer Res BCR. 2020 ;24(1):100. 10.1186/s13058-020-01339-x PMC7513536 32972455

[34] Chang CJ, Ish JL, Chang VC, Daniel M, Jones RR, White AJ. Exposure to per- and polyfluoroalkyl substances and breast cancer risk: a systematic review and meta-analysis of epidemiologic studies. Am J Epidemiol. 2024;193(8):1182–96. 38400646 10.1093/aje/kwae010 PMC11299034

[35] Yang XR, Figueroa JD, Falk RT, Zhang H, Pfeiffer RM, Hewitt SM, et al. Analysis of terminal duct lobular unit Involution in luminal A and basal breast cancers. Breast Cancer Res BCR. 2012;14(2):R64. 22513288 10.1186/bcr3170 PMC3446399

[36] Vierkant RA, Hartmann LC, Pankratz VS, Anderson SS, Radisky D, Frost MH, et al. Lobular involution: localized phenomenon or field effect? Breast Cancer Res Treat. 2009;117(1):193–6. 10.1007/s10549-008-0082-6 PMC2904055 18592369

