# Supplementary material for: Associations of perfluoroalkyl substances (PFAS) with terminal ductal lobular unit involution of the normal breast
Source: Breast Cancer Res. 2025 Aug 18;27:148. doi: 10.1186/s13058-025-02103-9 (PMC12363039; doi:10.1186/s13058-025-02103-9)
Supplement: Supplementary file 1 — Supplementary Material 1 [file 13058_2025_2103_MOESM1_ESM.docx]

**Supplemental Figure 1.** Plots generated by BKMR analyses exploring independent effects of PFASs mixture components on any observed TDLUs (yes *vs* no) (N=263).

**Supplemental Figure 2.** Plots generated by BKMR analysis to evaluate each PFASs mixture component per one SD increase when setting the other mixture components at 25^th^, 50^th^, or 75^th^ percentiles on any observed TDLUs (yes *vs* no) (N=263).

**Supplemental Figure 3.** Plot from BKMR analysis evaluating the overall effect of the PFAS mixture on the outcome of any observed TDLUs (yes *vs* no) (N=263).

**Supplemental Figure 4.** Plots from BKMR analysis exploring bivariate interactions between each component of the PFASs mixture on the outcome of any observed TDLUs (yes *vs* no) (N=263).

**Supplemental Figure 5.** Plots generated by BKMR analyses exploring independent effects of PFASs mixture components on number of observed TDLUs (N=263).

**Supplemental Figure 6.** Plots generated by BKMR analysis to evaluate each PFASs mixture component per one SD increase when setting the other mixture components at 25^th^, 50^th^, or 75^th^ percentiles on number of observed TDLUs (N=263).

**Supplemental Figure 7.** Plot from BKMR analysis evaluating the overall effect of the PFAS mixture on the outcome of number of observed TDLUs (N=263).

**Supplemental Figure 8.** Plots from BKMR analysis exploring bivariate interactions between each component of the PFASs mixture on the outcome of number of observed TDLUs (N=263).
